# Simultaneous electrophysiology and optogenetic perturbation of the same neurons in chronically implanted animals using μLED silicon probes

**DOI:** 10.1016/j.xpro.2023.102570

**Published:** 2023-09-19

**Authors:** Nathaniel R. Kinsky, Mihály Vöröslakos, Jose Roberto Lopez Ruiz, Laurel Watkins de Jong, Nathan Slager, Sam McKenzie, Euisik Yoon, Kamran Diba

**Affiliations:** 1 Department of Anesthesiology and Neuroscience Graduate Program, University of Michigan Medical School, Ann Arbor, MI 48109, USA; 2 Department of Electrical Engineering and Computer Science, University of Michigan, Ann Arbor, MI 48109, USA; 3 Neuroscience Institute, Langone Medical Center, New York University, New York, NY 10016, USA; 4 Department of Biomedical Engineering, University of Michigan, Ann Arbor, MI 48109, USA; 5 Department of Neuroscience, University of New Mexico, Albuquerque, NM 87131, USA; 6 Department of Mechanical Engineering, University of Michigan, Ann Arbor, MI 48109, USA; 7 Center for Nanomedicine, Institute for Basic Science (IBS) and Graduate Program of Nano Biomedical Engineering (Nano BME), Advanced Science Institute, Yonsei University, Seoul 03722, South Korea

**Keywords:** Neuroscience, Systems Biology, Biotechnology and Bioengineering

## Abstract

Micro-light-emitting-diode (μLED) silicon probes feature independently controllable miniature light-emitting-diodes (LEDs) embedded at several positions in each shank of a multi-shank probe, enabling temporally and spatially precise optogenetic neural circuit interrogation. Here, we present a protocol for performing causal and reproducible neural circuit manipulations in chronically implanted, freely moving animals. We describe steps for introducing optogenetic constructs, preparing and implanting a μLED probe, performing simultaneous *in vivo* electrophysiology with focal optogenetic perturbation, and recovering a probe following termination of an experiment.

For complete details on the use and execution of this protocol, please refer to Watkins de Jong et al. (2023).^1^

## Highlights

• Viral infusion of optogenetic construct to brain region of interest
• Probe preparation, mounting on a microdrive, μLED testing, and implantation
• Performing simultaneous optogenetics and electrophysiology with a μLED probe
• Probe recovery following experiment termination

## Abstract (editor highlights)

Publisher’s note: Undertaking any experimental protocol requires adherence to local institutional guidelines for laboratory safety and ethics.

## Graphical abstract

**Figure:**
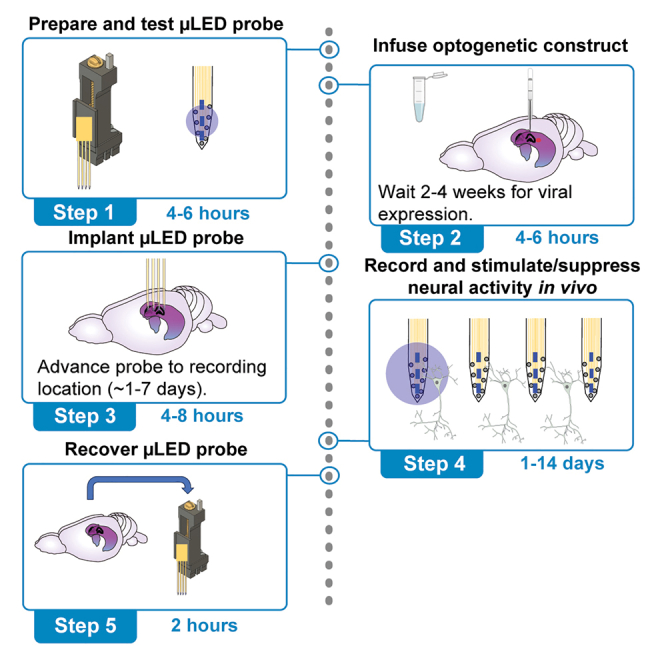

## Teaser

μLED silicon probes feature independently controllable miniature LEDs embedded at several positions in each shank of a multi-shank probe, enabling temporally and spatially precise optogenetic neural circuit interrogation. Here, we present a protocol for performing causal and reproducible neural circuit manipulations in chronically implanted, freely moving animals. We describe steps for introducing optogenetic constructs, preparing and implanting a μLED probe, performing simultaneous *in vivo* electrophysiology with focal optogenetic perturbation, and recovering a probe following termination of an experiment.

## Before you begin

Here, we provide a protocol that addresses all experimental stages for the reliable use of μLED probes. We begin with important considerations for choosing the appropriate optogenetic construct and how to deliver it to the region of interest. We next describe how to prepare the implant and eventually recover the μLED probe. Finally, we provide advice for *in vivo* neural excitation/suppression using optogenetics, troubleshooting steps, and best practices for post-processing of neural data. The steps below document the use of a μLED probe to stimulate neural activity in the rat hippocampus via photoactivation of virally induced channelrhodopsin. Nevertheless, μLED probes can also be used in a wide array of transgenic animal models and are compatible with other opsins whose absorption spectrum includes blue light. Note that this protocol is broadly applicable: along with providing μLED-specific guidance (see step-by-step methods details 2–20 below), it outlines many general principles important for performing chronic electrophysiological recordings with a wide variety of silicon probes.

Prior to testing and performing simultaneous electrophysiology and optogenetics, a μLED probe must be attached to a metal or plastic drive which is connected to a plastic stereotaxic holder. This will allow for post-implant adjustment of the depth of the probe.

### Institutional permissions

All procedures were performed in compliance with guidelines of the National Institutes of Health and approved by the University of Michigan’s Animal Care and Use Committee. Users of this protocol should obtain similar permission and guidance from the animal care committee at their institution(s).

### Build or purchase silicon probe microdrive

**Timing: 1–3 h, not including 3D printing time**

1. Build: Plastic 3D printed drive.
  a. 3D print drive parts for drives suitable for both mice and rats have been made publicly available. See Vöröslakos, Miyawaki et al. (2021)^2^ for further details and https://github.com/YoonGroupUmich/Microdrive for examples and different options.
  b. The drive should be put together following instructions provided by Vöröslakos, Miyawaki et al. (2021)^2^ or at https://github.com/YoonGroupUmich/Microdrive.
    ***Note:*** the designs listed here are optimized for use with μLED probes.
  c. However, drive designs can be modified and refined according to specific needs, for example by:
    i. shortening the drive base to accommodate implants to deeper brain regions, or,
    ii. widening the drive arm to accommodate higher density (non-μLED) probes.
  d. Inspect the open-source repositories listed above for the most up-to-date designs.
2. Purchase: Metal drive.
  a. Highly stable metal drives with a smaller footprint and similar weight to the plastic drives listed above are also available for purchase from 3DNeuro, along with drive holders.^3^
    ***Note:*** These drives cannot be customized unlike the plastic ones but offer a smaller footprint that can be helpful when space is limited, such as in mice.
  b. Additionally, the rigidity of metal allows for greater precision, durability, and reuse.
3. Print/build drive holder for plastic or metal drive (necessary files available at https://github.com/YoonGroupUmich/Microdrive) or purchase metal drive holder from 3DNeuro.

### Attach probe to drive

**Timing: 1–2 h**

Steps for attaching probes to metal and plastic drives are similar and are as follows.

4. Plastic drive: see Video 2 in Vöröslakos, Miyawaki et al. (2021)^2^ Video 2 for a video demonstrating the process of attaching probes to a plastic drive.
5. Metal drive: Key steps are included here.
  ***Note:*** See Methods video S1 for full visualization of the procedure for attaching probe to metal drives and Vöröslakos, Miyawaki et al. (2021)^2^ for further details.
  a. Position drive in probe holder, making sure it aligns with holder on all three axes.
  b. Set up helping hands or micromanipulators to guide probe and holder.
  c. Attach header pins to each side of the probe’s electronic interface board (EIB) with dental acrylic (Unifast Trad and Unifast Trad LC used here).
  d. Place the EIB vertically in one micromanipulator and the probe holder vertically in the other, aligning the drive arm with probe shanks and the EIB board.
  e. Maneuver the probe into close proximity with the drive arm, ensuring that both are aligned and that slack is maintained in the ribbon cable.
  f. Apply superglue to the drive arm and attach probe.
  i. Ensure by eye that probe shanks are aligned along all axes and adjust carefully with a toothpick or forceps as needed.
    **CRITICAL:** Implanting and driving a mis-aligned probe produces bending and shear stresses that significantly increase the likelihood of breaking probe shanks.
    ii. You will have 5–15 s of working time before the superglue cures.
    iii. Mis-aligned probes can be removed by soaking in acetone and re-attached later.
  g. After curing, move the EIB board close to header pins connected to the drive holder and solder additional header pins between the EIB and drive holder.
    i. Make sure all screws for detaching the probe holder from the stereotaxic arm and driving probe arm are visible. See Methods video S1.

Methods video S1. Attaching μLED probe to metal drive, related to before you begin steps 4-5

## Key resources table

**Table:** 

REAGENT or RESOURCE	SOURCE	IDENTIFIER
**Other**		

AAV1-hSyn1-SIO-stGtACR2-FusionRed	Addgene	105677
AAV5-CaMKIIa-hChR2(H134R)-EYFP	Addgene	26969
10-μL syringe	WPI	Nanofil
35 ga Nanofil beveled needle	WPI	NF35BV
Injection pump	Neurostar	Injection Robot
μLED probe	Neurolight or MINT program	N1-F21-O36/18
OSC1lite (12 channels)	MINT program	N/A
18-pin stimulation cable	MINT program or Neurolight	N/A
18-pin connector	Omnetics	PZN-18-DD
Stimulation cable wire	Cooner Wire	CZ 1187
HDMI breakout board	eLabGuy	HDMI-AF-BO-V2A
Cable jumper wires	DigiKey	1568-1644-ND
1028 ch recording system	Intan	RHS2000
32-channel headstage	Intan	C3314
Recording cable	Intan	Standard SPI cable
Metal microdrive	3D Neuro	R2Drive
Plastic microdrive and rat protective crown	Vöröslakos et al.^3^	https://doi.org/10.5281/zenodo.8209229
T-1 and T-2 Start Driver	Moody	2088 and 2089
Headstage tester	Plexon	HTU
High-precision stereotaxic instrument	Kopf	962
Stereotaxic electrode holder	Kopf	Model 1770
Antrin screws (for ground and reference)	Antrin (Fisher Scientific)	000-120 × 1/16 SL BIND MS SST
Ground and reference wires	Phoenix Wire	36744MHW-PTFE
Header pin	Digikey	PEC14SAGN
Mill max 0.05″ header connector	DigiKey	851-87-050-10-001101
Mill max 0.05″ receptacle/socket connector	DigiKey	851-43-050-10-001000
Multimeter	Tenma	72-9385
Drill bit (for viral infusion)	Fine Science Tools	19009–05
Trephine (for craniotomy)	Fine Science Tools	18004–23
1 mL syringe	BD	309659
18ga x 1 ½” needle	BD	305199
Curing Light	Henry Schein	CU 1000
1/16″ slotted electronics screwdriver	Mouser	247-614-2
C&B Metabond	Parkell (Henry Schein)	1865548EZ
Unifast LC (light cure acrylic)	Unifast	338021
Unifast Trad Ivory Powder + Liquid	Unifast	339104 and 339291
Dow sil	Dow	3-4680 Silicone Gel Kit
Vet wrap	3M	1404
32-channel headstage	Plexon	R
OmniPlex Neural Data Acquisition System	Plexon	N/A
Open Ephys Acquisition Board	Open Ephys	N/A
Neurolight Stimulator System	Plexon	N/A
Arduino	Arduino	Uno

**Software**		

OSC1lite Software	This paper	https://doi.org/10.5281/zenodo.8209373
RHX Recording Software	Intan	RHX
Open Ephys GUI	Open Ephys	GUI

## Step-by-step method details

### Introduce optogenetic construct to area of interest

**Timing: 2–3 h**

Here we outline general considerations for viral delivery of optogenetic constructs. The construct used and brain regions targeted will vary depending on the user’s experimental goals.

1. Select the appropriate virus for your brain region, species, and experimental question.
  a. The efficacy of virally induced protein expression can vary widely depending on the amount and titer of the virus injected, model species used, virus serotype, and targeted brain region.^4^^,^^5^
    **CRITICAL:** If you are using a new optogenetic construct, consider doing an expression study to determine how well your viral construct expresses the opsin in the desired species/brain region and assess the best dilution to obtain robust expression without overexpression or neurotoxicity.
    i. See Resendez et al. (2016)^6^ for dilution study methodology applicable to any protein of interest.
  b. Different constructs have different light sensitivities and absorption spectra.
  c. Additionally, many optogenetic constructs can alter intrinsic ion concentrations and biophysics after extended illumination^7^^,^^8^
  d. Note that presently μLEDs only produce blue light (centered emission at 460 nm), so make sure to choose an opsin that is activated by this wavelength of light.
    ***Note:*** The use of transgenic animals, such as the Thy1-ChR2-YFP mouse line, which expresses channelrhodopsin across multiple brain regions, can obviate the need for viral delivery altogether.^9^
2. Infuse virus of interest. See Methods video S2.
  a. Prepare the rodent for surgery using standard techniques and protocols approved by your institution.
    i. Perform a craniotomy just large enough to accommodate your infusion needle at the area of interest.
    ii. Stop any blood flow using pressure and cold, sterile saline or artificial cerebral fluid.
      ***Note:*** See^10^ and^11^ for details on anesthesia, scalp opening, skull preparation, etc.
  b. Carefully note any adjustments necessary to properly level and align the rodent in the stereotaxic apparatus.
    **CRITICAL:** Matching up your infusion and probe implant coordinates precisely is vital, and even small mismatches can result in failed experiments, particularly for deeper brain regions.
    i. This is mainly a concern when viral delivery and probe implant are performed on different days, typically 2–3 weeks later.
    ii. Always adjust the rodent’s nose as required during both infusion and implant procedures to ensure that the skull is level for each procedure.
    iii. If you find that the infusion craniotomy site is not readily visible when you attempt to perform your probe implants, you can score the skull surface in the shape of a plus or a circle (centered on the infusion site) with a dental drill at the time of the viral infusion to clearly mark your target skull areas.
  c. Load 1.5–2 μL of virus into a 10 μL syringe with a small gauge needle and affix the syringe to an automatic or manual injector attached to a stereotaxic arm.
    i. A 10 μL Nanofill syringe with a 35ga beveled needle and a Neurostar Injection Robot were used in this protocol, as shown in Methods video S2.
    ii. We typically perform three injections of 250 nL (50 nL/min) each at desired site and +/- 250 μm (total 750 nL) to maximize expression in our target area, but you may choose to inject other volumes of your construct.
      ***Note:*** The desired depth of infusion depends on the experiment and brain region of interest.
  d. Lower needle and infuse virus.
    i. Slowly lower needle or pipette to the lowest injection site, leave for 10 min to let settle.
    ii. Inject at 50 nL/min and wait 10 min to allow for diffusion.
    iii. Raise to next site and repeat steps i-ii until finished.
      ***Note:*** For deep brain regions, we recommend performing an additional injection of 250 nL 1–2 mm above your desired site (and outside of your brain region of interest).
    iv. This will allow you to verify that the optogenetic manipulation is working as desired while you are lowering the probe en route to the desired site.
3. Suture the craniotomy wound and provide post-operative care.
  **CRITICAL:** Typical wait times for full viral expression are 1–2 weeks for mice, 2–4 weeks for rats.
  a. For this reason, many experimenters choose to implant the probe at a later time point.

Methods video S2. Viral infusion of optogenetic construct, demonstrated on a 3D-printed plastic rat skull, related to step 2

### Test probe prior to implant

**Timing: 1 h**

This step is critical to verifying that all equipment required for μLED stimulation and recording are working properly prior to implant.

**Figure 1:**
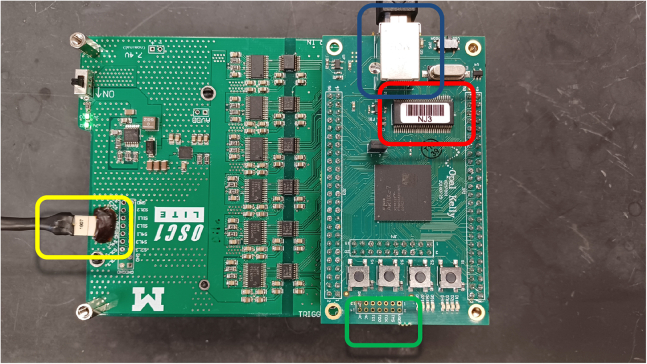
Cable Connections to OSC1lite 16 pin white stimulation cable shown plugged in on left side of board (yellow box). USB cable to computer shown at top (blue box). Note the “NJ3” identifier just below the USB connection port (red box). The TTLin ports are located on the side under the green box and the TTL out ports are located on the side under the USB connection (blue box).

**Figure 2:**
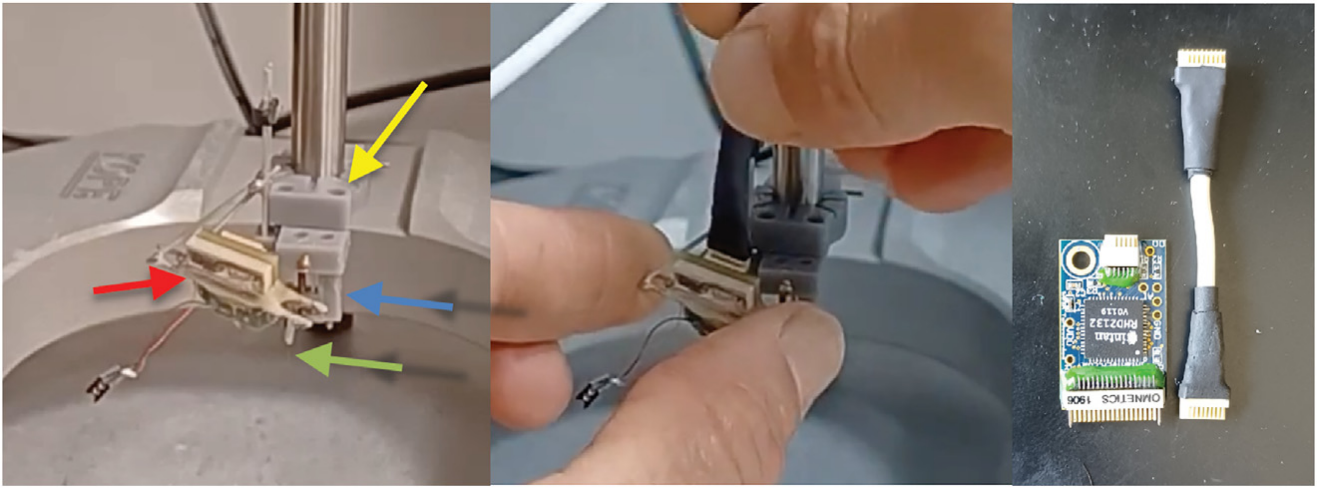
Probe testing configuration *Left*: μLED probe attached to plastic drive (blue arrow shows drive, green arrow shows drive arm), secured into a drive holder (yellow arrow), mounted on stereotaxic arm for testing prior to implant. The probe electronic interface board (EIB, red arrow) is secured to the drive holder by soldering header pins between the EIB and the probe holder. *Middle:* proper procedure for connecting stimulation cable, note fingers carefully supporting the μLED EIB during cable attachment. See also Figure 4 for a magnified view of the setup. *Right*: A cable stub can be constructed to allow the user to plug/unplug the stimulation cable above the level of the headstage in case of excessive cable twisting.

**Figure 3:**
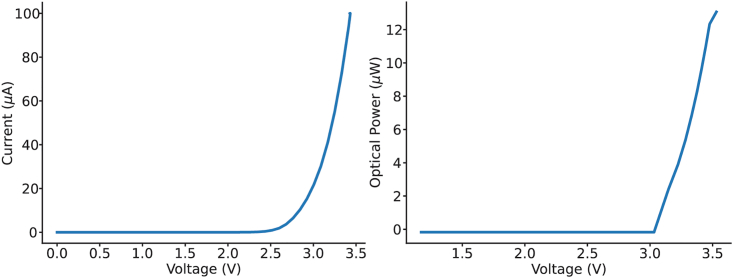
Example I-V and P-V curves for μLED 1 (L1) at the bottom of shank 3 Notice the sharp, non-linear increase in current and power at around 3V, highlighting the danger of using voltage drivers to activate μLEDs.

**Figure 4:**
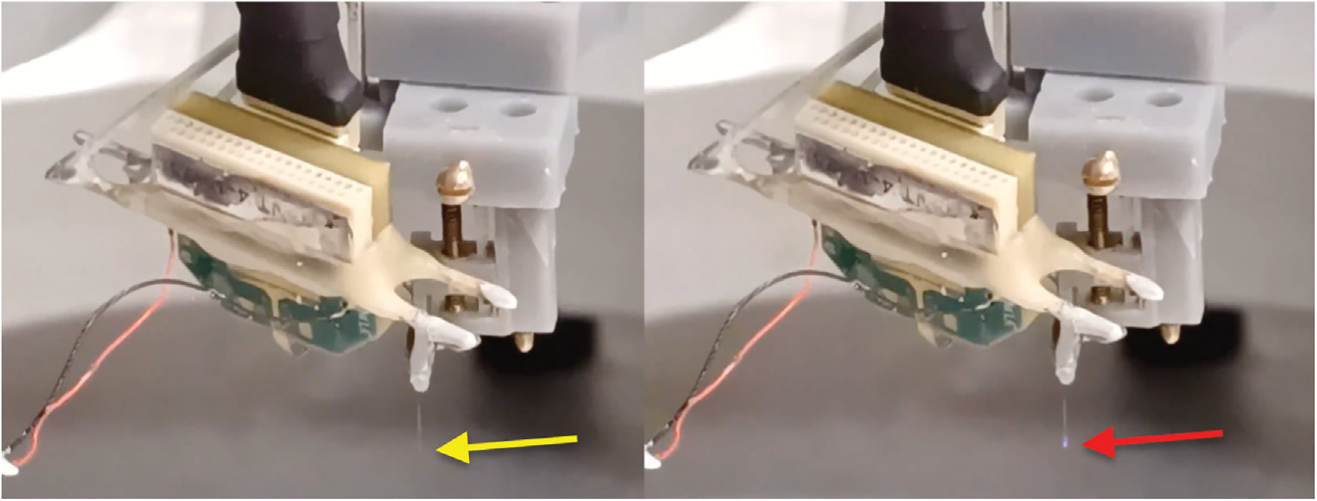
Example of proper LED activation by eye during testing *Left:* μLED is not active – current level is either too low to activate the light or μLED/OSC1lite software is not functioning properly (no blue light observable at tip of probe, yellow arrow). *Right:* Proper activation of a μLED by the OSC1lite software is indicated by a small amount of blue light visible by the naked eye (red arrow).

**Figure 5:**
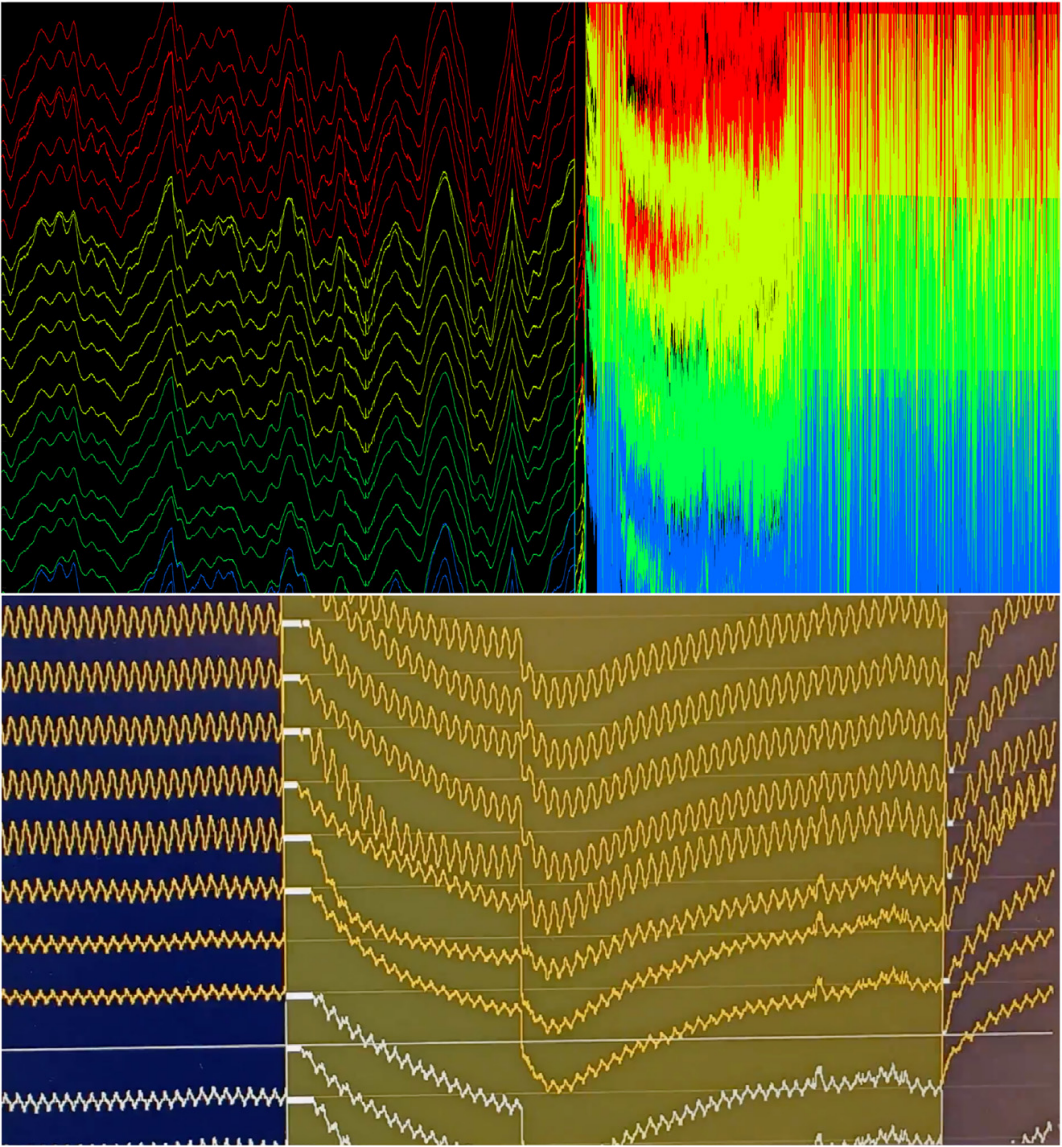
Example of stimulation artifacts (Top) Example *in vivo* stimulation artifact observed following activation of an excessively noisy μLED. An artifact of this magnitude should be identified during testing and the LED producing this effect should not be used. (Bottom) Example of an acceptable stimulation artifact observed while testing a μLED probe in saline *prior to* implantation. See also Methods video S4.

**Figure 6:**
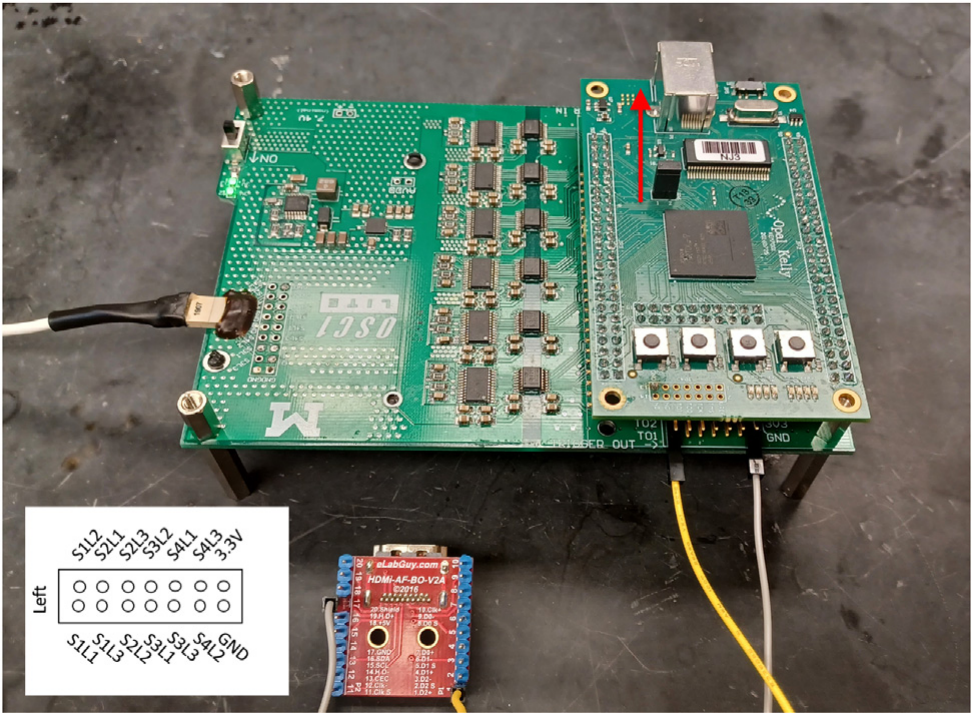
Example OSC1lite Trigger Out port connection In this case, the shank 1 LED1 (S1L1) μLED trigger out port (key = bottom right inset) is connected to the D2+ pin of an HDMI connector board with a yellow cable. The ground pin port is connected to the ground pin of the HDMI connector board with a gray cable. This system will be used to transfer a TTL for each activation of the S1L1 μLED to digital channel 1 of an OpenEphys acquisition system. Make sure the “Trigger Out” box (enabled by default) is checked for each pin you connect to in the OSC1lite software. Similar connections can be made for each μLED, and a similar strategy can be used to collect external triggers to activate μLEDs (same connection configuration on opposite side of the OSC1lite, red arrow). Be sure to select “External Trigger” under the “Trigger Source” column in the right side of the OSC1lite software for external triggering. Note that each μLED is triggered independently by an input TTL to the corresponding pin.

**Figure 7:**
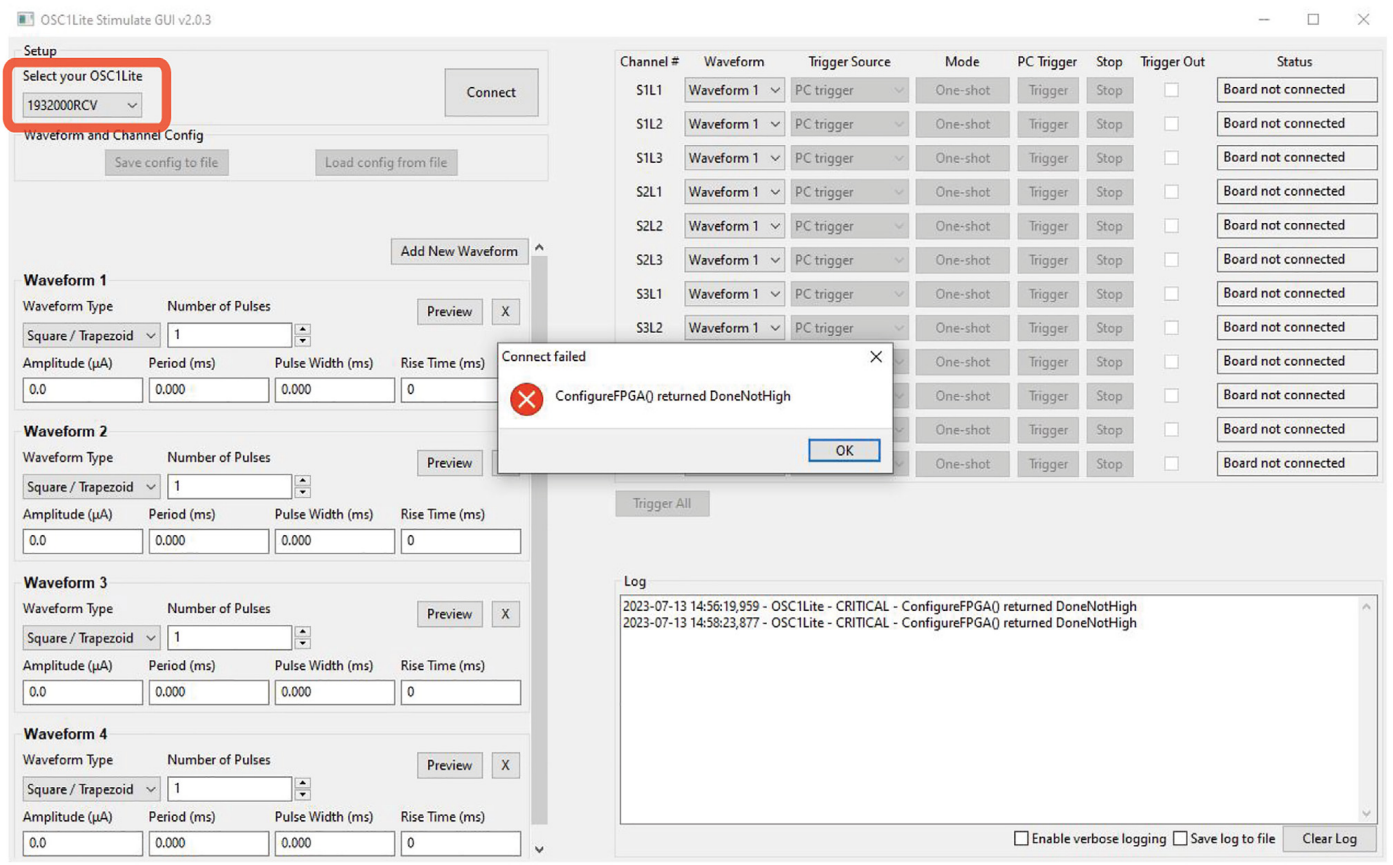
Incorrect selection of OSC1lite in software Note that the last three characters in the “Select your OSC1lite” dialog box shown in a red square (“FHF”) do not match the OSC1lite identifier “NJ3” (see Figure 1), resulting in a connection error.

**Figure 8:**
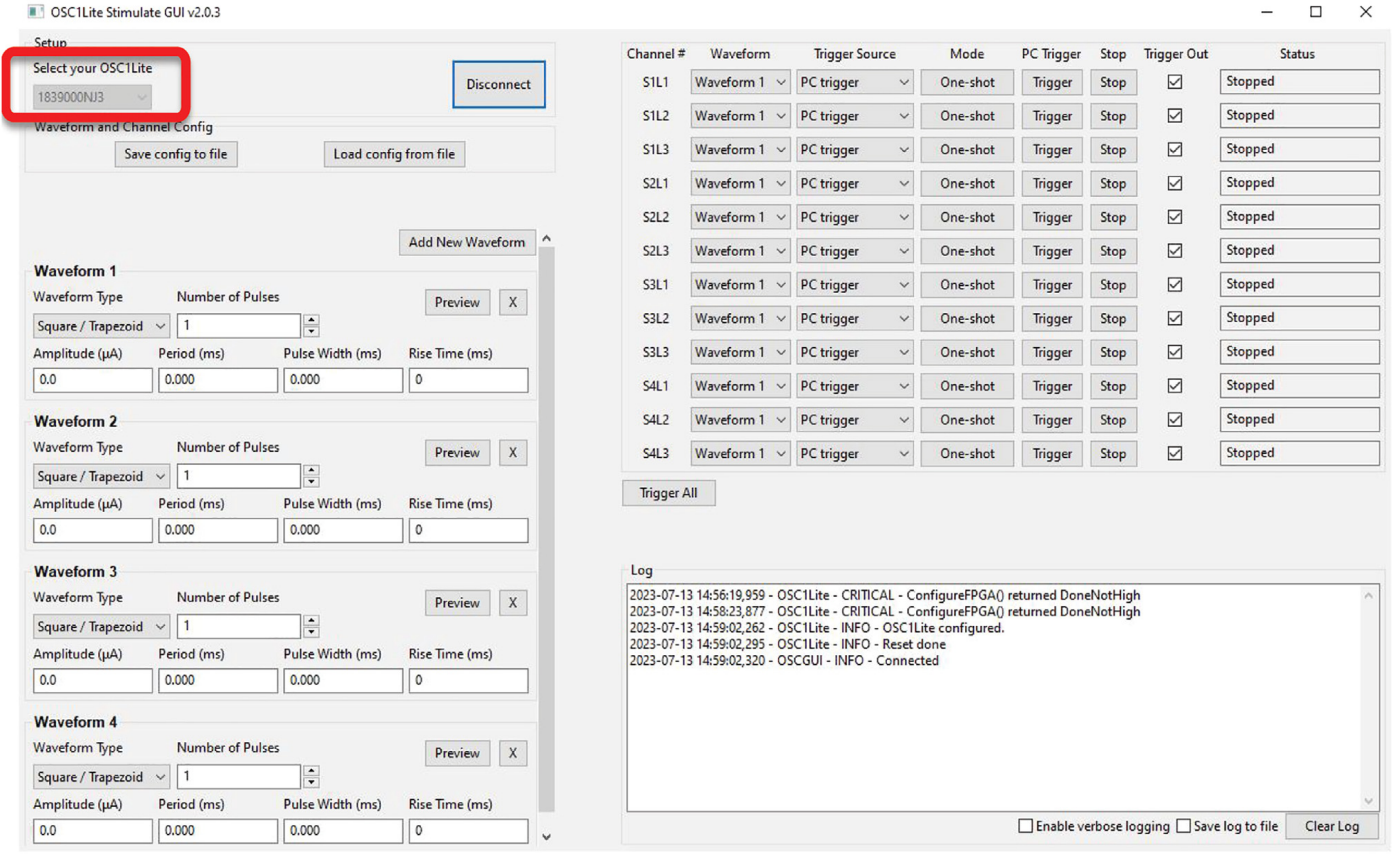
Correct connection to OSC1lite in software Note that last three digits under “Select your OSC1lite” (red box) match the “NJ3” identifier from Figure 1 resulting in a “Connected” printout to the Log in the lower right.

**Figure 9:**
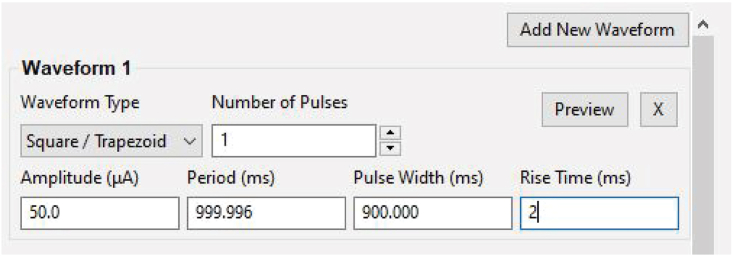
Waveform creation This figure shows how to create an example waveform which will deliver a single 50 μA pulse lasting 900ms with a 2ms rise time and 2ms fall time (at present, waveforms can only use the same rise and fall times) to mitigate stimulation artifact. Clicking “Preview” will plot out the waveform created as a visual check.

**Figure 10:**
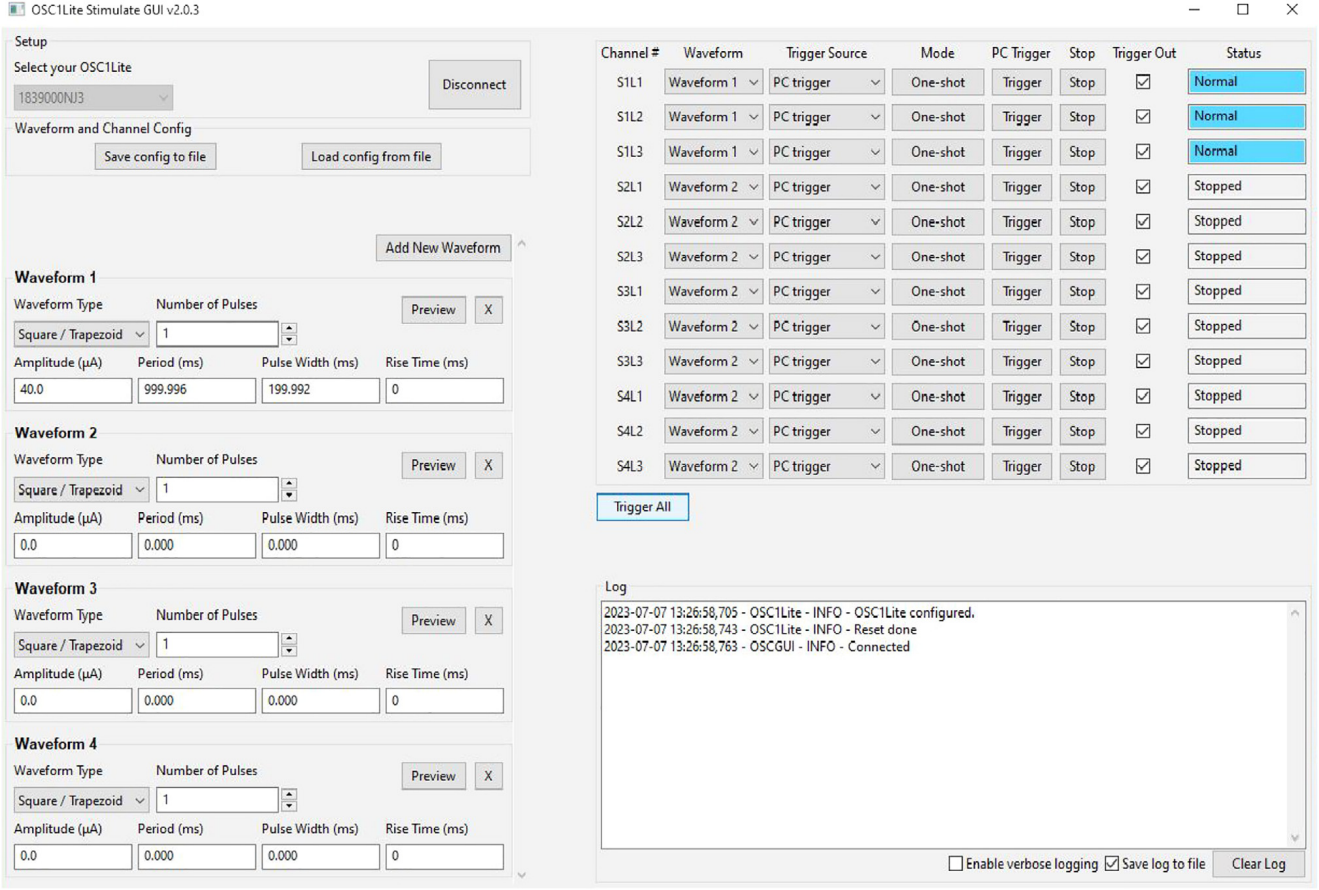
Example *in vivo* stimulation setup for focal stimulation of neurons near Shank 1 only Waveform 1 is set to deliver a 200ms, 40 μA pulse. Waveform 1 is applied to Shank 1 only on the right side of the software GUI such that only Shank 1 LEDs show up in blue as “Normal” during operation while the other LEDs, which are not activated (Amplitude = 0 μA), show up as “Stopped”.

**Figure 11:**
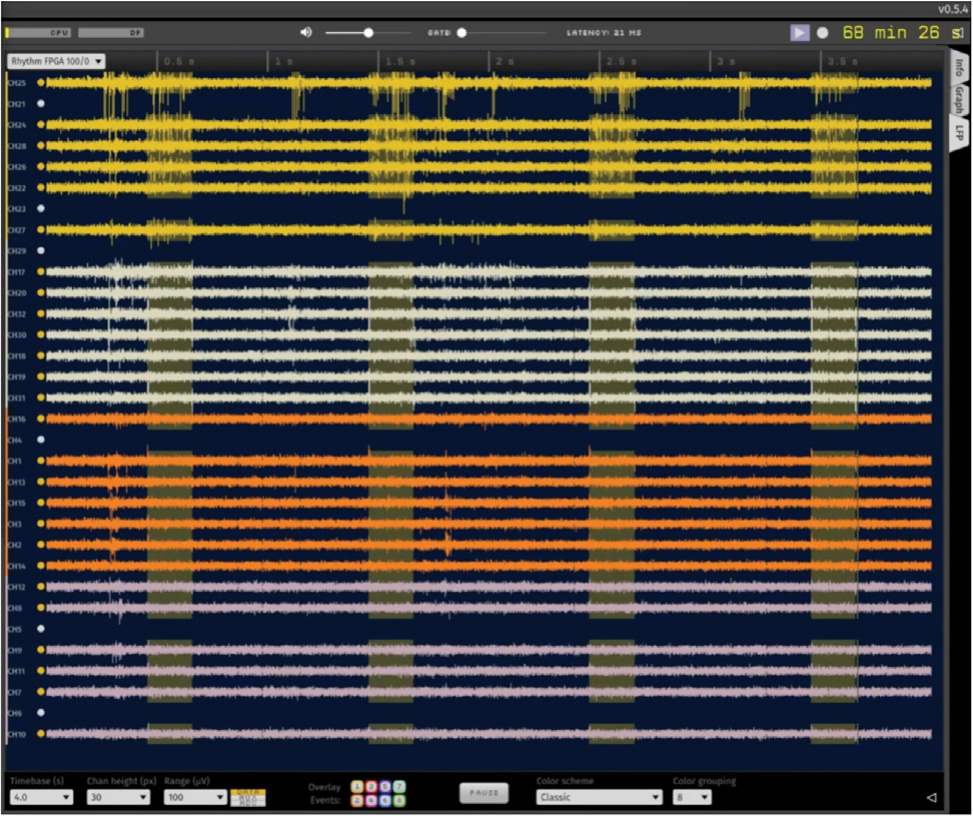
*In vivo* evoked spiking on Shank 1 using channelrhodopsin High pass filtered signal recorded with a chronically implanted μLED probe in a rat injected with AAV5-CaMKIIa-hChR2(H134R)-EYFP in region CA1 of the hippocampus (high impedance channels are not shown, n = 6 channels). Waveform 1 activation (see Figure 10) evokes robust spiking activity on shank 1 (yellow) but not on other shanks. Activation times are clearly shown in OpenEphys acquisition software as yellow vertical boxes, logged via sending the Shank 1 Trigger Out pins to Digital Input 1 (see Figure 6).

**Figure 12:**
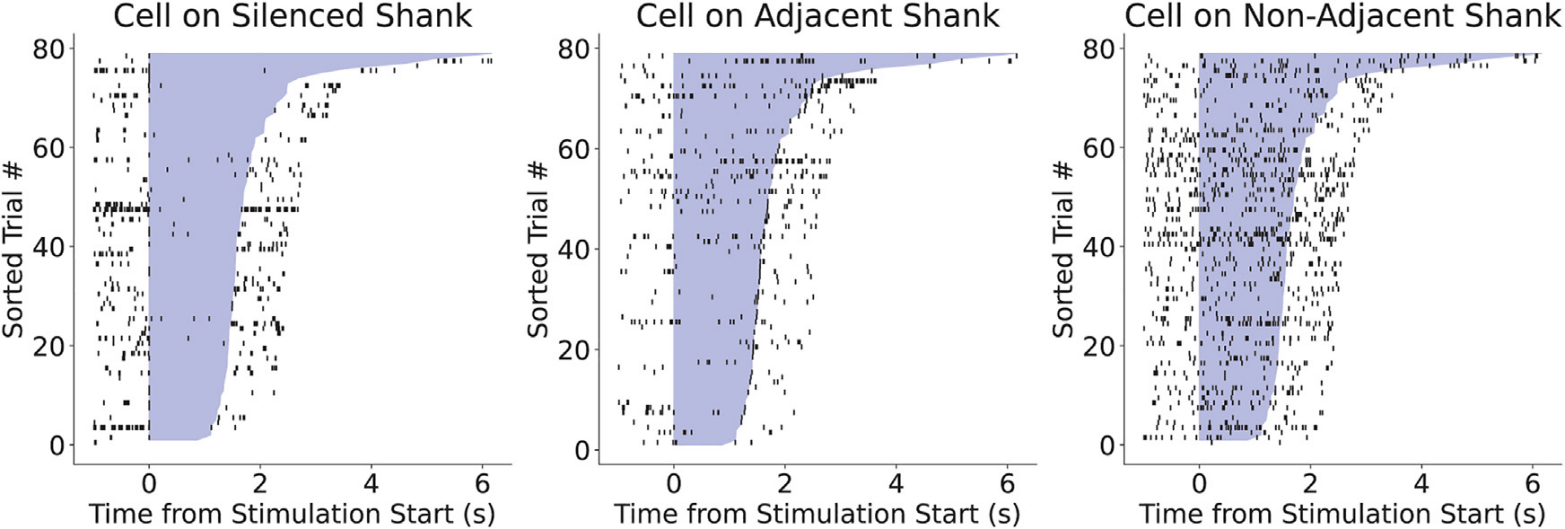
Example of successful, localized silencing of neural activity Raster plots of single units in the rat hippocampus during light application on Shank 1 μLEDs (left) only using the virally expressed inhibitory opsin StGtACR2 (AAV1-hSyn1-SIO-stGtACR2-FusionRed). Light was applied while the rat traversed the middle portion of a linear track, and trials are sorted from shortest light activation (bottom, rat running fastest) to longest light activation (top, rat running slowest). Note spiking activity before and after light application on shank 1. Mild suppression is still evident on the adjacent shank 2 (middle) while spiking activity remains un-perturbed on shank 3 (right).

4. Verify electrode impedances using Intan RHX recording software or other devices.
  a. Plug in 32-ch amplifier and connect to recording cable.
    **CRITICAL:** Hold the probe electronic interface board (EIB) carefully while plugging/un-plugging the headstage and stimulation cable to avoid breaking the extremely fragile probe shanks.
  b. Test electrode impedances using the Intan recording software or other devices.
    i. Submerge the mounted probe into saline, connecting a wire that is glued to the side of the container to the ground and reference wires of the probe.
    ii. Functional electrodes will have an impedance in the range of 1000–1500 kΩ.
      ***Note:*** impedances will drop following implantation in brain tissue.
5. Verify μLED functionality (see steps 13–20 below for options for powering μLEDs) using OSC1lite board and software. See Methods videos S3 and S4 and Figure 1, Figure 2, Figure 3, Figure 4, Figure 5, Figure 6, Figure 7 for setting up the OSC1lite and testing the μLED probe.
  ***Note:***Figure 1, Figure 2, Figure 3, Figure 4, Figure 5, Figure 6, Figure 7, Figure 8, Figure 9 illustrate key steps for setting up μLED probes for testing prior to implant or prior to use *in vivo*, while Figure 10, Figure 11, Figure 12 depict expected results during actual *in vivo* stimulation/silencing.
  a. Carefully plug in the stimulation cable to the OSC1lite (Figure 1) and probe electronic interface board (EIB) (Figure 2).
    i. See “Stimulate/silence neural spiking activity with OSC1lite” section below (steps 13-19) for more detailed instructions on using the OSC1lite.
      **CRITICAL:** We recommend attaching the probe to a stereotaxic arm to avoid breakage during testing.
  b. Consult the manufacturer-provided I-V curves and provide current at just below and just above the onset current to verify μLED activation.
    **CRITICAL:** Do not exceed 100 μA to avoid burning out the μLEDs.
    **CRITICAL:** While you can use a voltage-controlled waveform generator to power the μLEDs, we do not recommend doing so due to the non-linearity of the I-V curve.
    i. If you do use a voltage generator, be especially careful to keep your voltage at levels well below the max shown on the I-V curve and/or use a series resistor to limit your current output (Figure 3).
  c. Visually verify activation of each μLED (can be done with the naked eye or under a surgical scope)
    i. Take note of possible differences in activation threshold between μLEDs (Figure 4 and Methods video S4).
      ***Note:*** the typical irradiance of each μLED operating at max power of 100 μA is 33 mW/mm^2^.
      ***Note:*** μLEDs can sometimes induce electrical noise during activation.
    ii. To test for noise, we recommend visualizing electrical activity with the probe submerged in saline (see 4.b) while activating each μLED independently.
    iii. See also Methods video S4 and Figure 5 for examples of acceptable vs. unacceptable artifact.

Methods video S3. Setting up OSC1lite and preparing μLED probe for testing, related to steps 5, and 13–17

Methods video S4. Testing μLED probe prior to implant with OSC1lite, related to step 5

### Implant μLED probe

**Timing: 3–6 h**

Procedures for implanting silicon probes have been detailed elsewhere: we recommend following Vandecasteele et al. (2012)^11^ for a comprehensive protocol covering probe implant for chronic, *in vivo* recording in freely moving rodents.

6. See Methods videos S5 and S6 for an example rat implant procedure in accordance with Vandecasteele et al. (2012)^11^ and Vöröslakos et al. (2021).^3^.

**CRITICAL:** We highly recommend using separate screws for ground and reference wires to help mitigate potential issues with line noise (see troubleshooting).

7. Here we provide μLED-specific recommendations for users to consider based on their brain region of interest, which will influence drive design and implant.
  a. To provide maximum flexibility, e.g., for a user’s first implant or when implanting multiple probes in the same animal, we recommend using a plastic crown base with copper mesh for implant protection, as described in Vöröslakos et al. (2021).^2^
  b. Once the process for a single implant is streamlined, we recommend using a hybrid plastic/mesh cap for mice or a fully plastic cap for rats, as described in Vöröslakos et al. (2021).^3^

***Note:*** Be aware that the application of blue light to cure epoxies used in these procedures can potentially penetrate the skull and photoactivate blue-light opsins compatible with μLED probes such as channelrhodopsin or stGtACR2.

8. Key implant steps (See Methods videos S5 and S6).
  a. Secure anesthetized animal with stereotaxic instrument ear bars and prepare skull for surgery in accordance with your approved protocol.
  b. Level skull and mark coordinates for probe implant. These should match your infusion coordinates (see 2.b above).
  c. Cover remainder of skull with a thin layer of translucent Metabond to prepare it for attaching the plastic crown base.
  d. Insert ground and reference screws (above the cerebellum in our experiments) with wires attached.
    i. You can either leave the wires bare and solder them directly to the corresponding probe wires or solder them to a Mill-Max connector for easy attachment to probe wires.
  e. Attach the 3-D printed plastic crown base to the skull with dental acrylic on inside and outside edges of crown.
    ***Note:*** We recommend printing out several slightly different shapes in advance to allow you to select the one that best fits your craniotomy.
  f. Perform craniotomy with a burr and drill set.
    i. We recommend using a 1.8 mm trephine burr for simplicity.
    ii. Remove dura and stop any bleeding by applying cold sterile saline or artificial cerebral fluid which will also protect the brain surface from drying out.
  g. Attach probe and probe holder to stereotaxic arm and lower to the brain surface.
    i. Make sure probe shanks are protruding below bottom of the microdrive base.
    ii. if not, turn top flat-head screw to lower shanks as needed, taking care not to push the drive out of the holder.
  h. Slowly lower probe to ∼0.5 mm above your region of interest (actual implant depth can vary depending on experimental and anatomical constraints).
    **CRITICAL:** Watch carefully under a microscope while lowering the probe to make sure shanks do not buckle upon insertion through the pia or remaining dura.
    i. If they begin to bend, raise up, inspect brain surface for dura that could be impeding probe penetration, and lower again.
    ii. The pia will typically give way after two or three attempts.
    iii. Alternately, it may be scored with a needle tip, but be careful not to cause excessive tissue damage.
  i. Secure probe base to skull with dental acrylic. Do not let acrylic flow between the drive base and the drive or into the craniotomy and onto the probe.
  j. Cover the exposed brain surface with sterile wax to protect brain and probe.
    i. Place small bits of wax near the craniotomy and by moving the soldering iron close to (but not touching) the wax and allow it to melt into position around the probe shanks.
    ii. Dow-sil can also be added prior to bone wax to provide a thin layer over the dura that the probe can still penetrate.
      ***Note:*** Avoid use of stiffer silicone elastomers such as Kwik-sil, which may impede movement of the probe and result in broken shanks.
  k. Unscrew top screws from stereotaxic attachment to drive holder and carefully retract stereotaxic arm, being sure not to bump the holder.
  l. Attach header pin to electrode holder on other stereotaxic arm, maneuver close to the EIB, and solder pin to the EIB.
  m. Attach crown sides to the crown base by lowering over base and tightening the front crown screw. This will allow the crown sides to swivel into position for attaching the EIB.
  n. Use stereotaxic arm to maneuver the EIB to align with support pins on the side of the crown and solder each side of the EIB to support pins.
  o. De-solder the EIB from the stereotaxic holder pin and retract.
  p. Loosen the side set screw from the probe holder and remove.
  q. Steps k-p will work for any implant and will provide additional clearance for reinforcing the drive base connection and applying Dow-sil if required.
  r. Alternatively, for single-drive implants with plenty of clearance, users can perform steps as follows:
    i. Attach the EIB to the header pin on the second stereotaxic arm.
    ii. De-solder the EIB from the probe holder.
    iii. Unscrew the side screw.
    iv. Raise up the probe holder using the stereotaxic arm and perform steps m-o
  s. Attach ground and reference wires from the skull to the matching wires on the EIB either by soldering or by using mating Mill-Max connectors.
    i. Tuck wires away from probe shank, and if using Mill-Max connectors, secure them to the crown base with acrylic.
  t. Close crown and tighten two sideways screws on back.
  u. Cover the crown with the plastic cover and apply Vetwrap around the sides, securing with lab tape or a Velcro cable strap.
  v. Perform any final surgical steps (e.g., applying topical antibiotics) per your approved protocol, withdraw rodent from anesthesia, and begin post-operative monitoring.
9. Provide care to the animal and allow it to recover from the surgery in accordance with your approved protocol.

Methods video S5. Preparation steps prior to implanting a μLED probe, including ground and reference wire attachment and plastic crown base attachment to skull, shown on a 3D-printed plastic rat skull, related to step 8.a-f

Methods video S6. Implanting μLED for chronic recording in rat, illustrated on a 3D-printed plastic rat skull with plastic crown base and ground/reference screw attached, related to step 8

### Lower probe to region of interest

**Timing: 5–7 days**

Prior to performing optogenetic stimulation or suppression, position your probe into the proper location.

10. To ensure good behavior during your planned experiments, is important to habituate the animal to the recording cable and related procedures during its recovery period.
11. Starting the day following implant, gradually lower the probe drive down to the target region, by turning the screw on the microdrive.
  a. Keep track of the number of screw turns you perform in order to maintain an estimate the probe depth (280 μm per turn for drives listed above in section 2).
    ***Note:*** We recommend verifying the estimated probe location against electrophysiological signatures specific to the brain region targeted by the probe.
  b. For optimal stability, do not exceed 140–280 μm each day (0.5–1 turn).
    i. Further reduce to 70–140 μm/day as you approach the region of interest.
    ii. To allow the brain and probe to settle, and to prevent breaking the probe, do not perform more than ¼ to ½ turn every 30 min.
      **CRITICAL:** you should observe a change in the observed neural signal immediately upon turning the screw, followed by a slower change over the next several hours as the brain tissue settles around the probe.
    iii. As this can be a non-linear process, we recommend waiting at least 6–8 h before each full turn of the screw to ensure that you don’t accidentally drive the probe past the target region.
12. **[Optional]** If you are performing deep brain recordings, we recommend testing the μLEDs in superficial brain regions as you advance the electrodes in order to determine initial illumination settings and identify/troubleshoot any issues prior to reaching your target region.
  a. It is also important to note that even focused manipulations/perturbations can affect downstream circuits, so be sure to consider anatomical and functional connectivity with your region of interest prior to performing and test perturbations.
  b. See the following steps 13-18 for full details.

### Stimulate/silence neural spiking activity with OSC1lite

**Timing: 1–2 h**

Here we outline the procedure for performing simultaneous optogenetics and electrophysiological recordings *in vivo* using an OSC1lite stimulation system to manually or automatically activate μLEDs.

13. Follow all directions for operating the OSC1lite provided at: https://github.com/YoonGroupUmich/osc1lite.
14. Tape stimulation and recording cables together to keep tidy and facilitate disconnection/reconnection, which may be necessary in case of excessive cable twisting during experimentation.
15. **[Optional]** Set up the OSC1lite to interface with external equipment.
  a. Connect TTL output pins on the OSC1lite to event/TTL ports on your recording system. See Figure 6 and Methods video S3.
    i. Female to female cable jumpers and an HDMI breakout board are used to interface with an OpenEphys acquisition system in Methods video S3.
      ***Note:*** this will denote TTL on/off trigger times in your recording file but will not record waveform parameters – users should make note of the waveforms they use in their experimental log.
  b. If performing closed-loop or automated, open-loop stimulation/silencing, connect TTL input pins on the OSC1lite to hardware supplying the triggering signal.
16. Initialize the OSC1lite to probe connection. See Methods video S3.
  a. Power on the OSC1lite.
  b. Connect to open-source OSC1lite software and make sure all channels are off. Make sure you select the device whose last three characters match the identifying characters on your OSC1lite board (Figures 7 and 8).
  c. Connect an 18-pin stimulation cable first to the board, then to the probe (Figure 2). Custom cables can be manufactured using Omnetics PZN-18-DD connectors. d. Connect the OSC1lite ground to the recording system ground.
  e. Attach a 32 channel headstage to the probe.
  f. Connect a recording cable (12-pin SPI for Intan) to the recording system and then to the headstage. See Methods video S7 for cable/headstage attachment and Methods video S8 for detachment following recording.
    ***Note:*** the headstage will block access to all but one side of the stimulation cable connector on the probe. Therefore, we recommend connecting the stimulation cable first.
  g. One strategy to deal with excessive cable twisting (e.g., especially in recordings involving rats rather than mice) is to build a short 18-pin cable “stub,” approximately the length of the headstage to facilitate cable disconnection/reconnection in the middle of a recording (Figure 2, right). A cable stub can be built as follows.
    i. Secure two Omnetics 18 pin connectors (PZN-18-DD) in helping hands.
    ii. Solder 18 short (∼2.0 cm) insulated wires (Cooner Wire CZ 1187) between connectors.
    iii. Cover all wires/connections with heat shrink.
    iv. See Figure 2 for picture of final assembled product.
17. Titrate light levels for photo stimulation. See Methods video S9.
  a. We recommend performing these steps in the animal’s home cage or a dedicated rest box prior to experiments.
  b. Create your desired stimulation waveform in the OSC1lite GUI and set appropriate minimum light levels for each μLED based on the probe testing protocol outlined in steps 4-5. See Figure 9.
    i. We recommend starting with a square waveform with 2ms rise time, 100ms pulse width, 2 s period, and 10 pulses (Figure 10).
    ii. This will give you sufficient trials to evaluate the efficacy of stimulation/silencing at a given light level.
  c. Trigger each μLED manually while recording.
    i. Adjust the light level up/down by adjusting the waveform amplitude for each μLED, as your experiment requires, to provide the lowest light power necessary for the required level of optogenetic stimulation/suppression.
    ii. See Figure 11 for an example of a successful stimulation of neurons located near shank 1.
    iii. Make note of the light level for each μLED and save these as separate waveforms in the OSC1lite software. See Figure 9.
  d. Create waveforms for each μLED based on titration results.
    **CRITICAL:** Be sure to choose the appropriate waveform, trigger source (PC or External), and Mode (One-shot or Continuous) for each μLED in the rightmost window of the OSC1lite software. See Figures 9 and 10.
    i. Check "Trigger Out" for each μLED whose activation you want to record in the recording software.
    ii. Note that L1 (the bottom-most μLED on each shank) is shown at the top of the software window even though it is physically located at the bottom of each shank.
18. Stimulate/Silence. See Methods video S9.
  a. Option 1: Trigger manually
    i. Click "PC Trigger" for individual μLEDs or "Trigger All" to illuminate all μLEDs simultaneously.
  b. Option 2: Trigger externally (for automated or closed-loop designs)
    i. Select "External Trigger" in the right window.
    ii. Send TTL to the OSC1lite TTLin port(s) from external hardware.
    iii. Note that the OSC1lite will apply the appropriate waveform at the onset of each external TTL and that the full waveform will then occur, which will not necessarily match the length of the external TTL.
19. Patterned stimulation (Vöröslakos et al., 2022).^12^
  a. For more complex signals, you can use an Arduino to trigger specific μLEDs using the TTL inputs of OSC1Lite.
  b. Set Trigger to external mode, specify current amplitude, duration, and duty cycle.
    ***Note:*** This preconfigured stimulation waveform will be triggered every time the OSC1Lite receives a TTL input.
  c. To test the pattern, you may use a Plexon Headstage Tester Unit.
20. Alternative μLED powering options.
  a. μLEDs can also be powered with custom-built current driver circuits (e.g., Stark et al., 2012) or an on-head current driver design (Tarnavsky Eitan et al., 2021).
  b. Plexon’s NeuroLight Stimulator System is a commercially available μLED current driver.
  c. A voltage driver.
    **CRITICAL:** Voltage driver words of caution. While constructing a simple voltage driver or using a waveform generator to activate μLEDs is possible, we discourage this and include a discussion here as a word of caution.
    i. While the OSC1 lite and other current driver circuits, such as the one listed above in 20.a and 20.b, limit the current passing through a μLED, current and power can rise in a sharp, non-linear fashion with small increases in voltage when current is not restricted.
    ii. This can easily overpower and burn out a μLED (see Figure 3).
    iii. Nevertheless, we note that several studies have used voltage drivers to power μLEDs apparently without incidence.^13^

Methods video S7. Plugging in headstage, recording cable, and stimulation cable for chronic recordings and stimulation, relate to steps 16.e-16.f

Methods video S8. Detaching headstage and cables after recording, related to steps 16.e-16.f

Methods video S9. Example of successful *in vivo* activation of neurons on individual shanks in region CA1 of the rat hippocampus using channelrhodopsin, related to steps 17-18

### Probe recovery and post-recovery care

**Timing: 1–2 h**

Once the experiments are finished, μLED probes can be recovered and re-used in future experiments. Fundamentally, the procedure for performing a probe recovery is similar to that of implanting a probe, with the steps reversed. Here we note the key steps for successful probe recovery, which are also demonstrated in Methods video S10. Full details of probe recovery and cleaning steps can also be found in Vöröslakos et al. (2021).^3^

21. Prepare rodent for probe recovery.
  a. Anesthetize rodent per your approved protocol and secure in stereotaxic frame.
  b. Level skull.
  c. Position two stereotaxic arms near the crown, one with an electrode holder and clip attached, the other with the drive holder used for the implant.
22. Attach the probe EIB to the drive holder.
  a. Secure the header pin vertically in the electrode holder and maneuver close to the EIB board on the crown.
  b. Carefully loosen side screws on the crown and open the crown side to which the EIB is NOT attached, so as to provide clearance.
  c. Make sure to maintain slack in the ribbon cable to avoid damaging it.
  d. Solder the header pin from the electrode holder to the EIB.
  e. De-solder the EIB from crown support pins and move/rotate the EIB with the stereotaxic arm to make room for the probe holder.
23. Secure probe drive in drive holder.
  a. Maneuver the drive holder into position and align visually along all three axes by rotating the holder and/or adjusting the height of the nose cone.
  b. Carefully and slowly lower the drive holder onto the top of the drive, taking care not to impart any lateral force on the drive (which could break the probe).
  c. Tighten the set screw on the side of drive holder by hand or with a hex key.
  d. Maneuver the EIB close to the drive holder and use solder to support the header pins on the drive holder.
  e. De-solder the EIB from the pin on the electrode holder and retract/remove the electrode holder.
24. Remove probe and drive from animal.
  a. Loosen the bottom set screw securing the drive to the drive base with a T1 star screwdriver.
  b. Raise up the stereotaxic arm slowly, making sure that the drive moves up smoothly.
    **CRITICAL:** The drive may not move if it is poorly aligned to the drive holder or if there is acrylic between the drive base and drive. If the drive does not move, perform the following steps.
    i. Carefully remove any acrylic near the drive/drive base interface using forceps.
    ii. Re-tighten the T1 screw connecting the drive to the drive base.
    iii. Loosen the side set screw.
    iv. Raise up the drive holder.
    v. Carefully re-align. This may require applying small adjustments to the skull level.
    vi. Repeat steps 23–24b.
25. Remove probe from stereotaxic arm and attach to helping hand to submerge into cleaning solution.
  a. Note that using an enzymatic cleaner such as Ultrazyme is recommended as other cleaning agents can compromise the integrity of μLEDs and electrodes.
26. Be sure to re-test the probe electrode impedances and μLED functionality prior to re-use. See steps 4-5 above.

Methods video S10. Example probe recovery of probe implanted on 3D-printed rat skull, shown in Methods video S6, related to steps 22-25

## Expected outcomes

Representative results from successful, focal stimulation of neural activity in the rat hippocampus *in vivo* are shown in Methods video S9, Figure 11 and in published studies.^1^^,^^12^^,^^13^^,^^14^^,^^15^^,^^16^^,^^17^ An example of successful neural suppression is shown in Figure 12 for the rat hippocampus. Following the above protocol will ensure a high probability of successful neural perturbation with μLED probes. Based on our experience with the hippocampus, successful focal silencing using all μLEDs on one of the edge-most shanks should result in almost complete neural suppression on that shank, partial inhibition of spiking on the adjacent shank, and no disruption of activity on the furthest shank away (Figure 12). Likewise, successful photoactivation should induce spiking on one shank only and have little to no effect on spiking on adjacent shanks (Figure 11). Note that the extent of an optogenetic perturbation can vary depending on local microcircuit connectivity in the region of interest, opsin type/expression patterns in cell and cell subcompartments, and μLED location relative to neurons. These factors can result in excitation or suppression that spreads more strongly to neighboring shanks than shown in Figures 11 and 12, or even in paradoxical increases/decreases in firing which may indicate inhibitory stabilization.^1^ See also Methods video S9 for an example of successful, focal stimulation of neural activity on individual shanks in region CA1 of the rat hippocampus *in vivo.*

## Limitations

μLED probes are designed to provide focal delivery of blue light that allows for manipulation of neurons in close proximity to each μLED, with typical irradiance of 33 mW/mm^2^ at maximum operating current. As such, μLED probes are not designed for large scale stimulation or silencing of entire brain regions and are currently only compatible with blue-light sensitive opsins. Custom-build optrodes with light delivery via large diameter optical fibers^1^^,^^18^^,^^19^ may be better suited for such large scale stimulation/silencing, such as to test the role of a specific group of neurons in a given task (though even small numbers of neurons may prove sufficiency/necessity^20^).

The major advantages of μLED probes, as compared to simple use of optical fibers or low-channel optrodes, are 1) that they allow for the monitoring of neuronal activity both at the site of photostimulation as well as site further away from the light source and 2) that the integration of the μLED on the probe shank delivers an extremely low profile that minimizes damage to surrounding tissue, in contrast to the substantial diameters required in optical fibers. It is also worth repeating that complex interactions within neuronal circuits and inhibition stabilization can produce unexpected activities at and away from photostimulation sites, which may be important for proper interpretation of the results of these manipulations.^1^

## Troubleshooting

On occasion little silencing or photoactivation may be evident during light stimulation. The two most frequent causes of failed optogenetic perturbation are 1) improperly functioning μLEDs, and 2) a lack of opsin expression near the recording site.

### Problem 1: Improperly functioning LEDs

Carefully following all steps listed above will help identify any improperly functioning or noisy LEDs prior to performing an experiment.

### Potential solutions

• Inject opsin above the region of interest for testing and troubleshoot prior to recording.
• Carefully test all electrodes prior to implant.
• Check all μLEDs prior to implant. See Methods video S4 and Figure 5 for illustration of acceptable stimulation artifact during μLED activation.

### Problem 2: Lack of opsin expression near the recording site

The most common causes of failed stimulation/suppression are poor viral expression of the opsin or a mismatch between the opsin expression site and implant location.

### Potential solutions

• Perform a study prior to your experiment to verify adequate viral expression.
• Perform multiple viral injections at and above/below your intended stimulation site.
• Use transgenic animals.
• Perform careful histological verification following failed stimulation/silencing to determine where mis-targeting errors arise.

### Problem 3: Line noise or other high frequency noise

In some cases, connection of the stimulation cable can induce line noise or other high frequency noise.

### Potential solution

Separating ground and reference wires will help mitigate these noise issues. **Note**, however, that ground and reference channels are shorted by default on the standard 32 channel Intan RHD headstage. Therefore, if you are using this headstage in your recordings, you will also need to manually remove the 0-Ω resistor connecting the two channels to separate the ground and reference.

## Resource availability

### Lead contact

Further information and requests for resources and reagents should be directed to and will be fulfilled by the lead contact, Kamran Diba, kdiba@umich.edu.

### Materials availability

No newly generated materials are associated with this protocol. As listed in the key resources table above, μLED probes are available through the U-M MINT program from Plexon, and metal drives can be purchased from 3DNeuro or can be built according to these instructions: https://buzsakilab.github.io/3d_print_designs/.

### Data and code availability

Code and files for 3d printing and constructing plastic drives, drive holders, and plastic protective crowns used in this study can be found at https://doi.org/10.5281/zenodo.8209229. OSC1lite software used in this protocol can be found at https://doi.org/10.5281/zenodo.8209373.

## References

[1] Watkins de Jong L., Nejag M.M., Yoon E., Cheng S., Diba K. (2023). Optogenetics reveals paradoxical network stabilizations in hippocampal CA1 and CA3. Curr. Biol..

[2] Vöröslakos M., Miyawaki H., Royer S., Diba K., Yoon E., Petersen P., Buzsáki G. (2021). 3D-printed Recoverable Microdrive and Base Plate System for Rodent Electrophysiology. Bio. Protoc..

[3] Vöröslakos M., Petersen P.C., Vöröslakos B., Buzsáki G. (2021). Metal microdrive and head cap system for silicon probe recovery in freely moving rodent. Elife.

[4] Aschauer D.F., Kreuz S., Rumpel S. (2013). Analysis of Transduction Efficiency, Tropism and Axonal Transport of AAV Serotypes 1, 2, 5, 6, 8 and 9 in the Mouse Brain. PLoS One.

[5] Burger C., Gorbatyuk O.S., Velardo M.J., Peden C.S., Williams P., Zolotukhin S., Reier P.J., Mandel R.J., Muzyczka N. (2004). Recombinant AAV viral vectors pseudotyped with viral capsids from serotypes 1, 2, and 5 display differential efficiency and cell tropism after delivery to different regions of the central nervous system. Mol. Ther..

[6] Resendez S.L., Jennings J.H., Ung R.L., Namboodiri V.M.K., Zhou Z.C., Otis J.M., Nomura H., McHenry J.A., Kosyk O., Stuber G.D. (2016). Visualization of cortical, subcortical and deep brain neural circuit dynamics during naturalistic mammalian behavior with head-mounted microscopes and chronically implanted lenses. Nat. Protoc..

[7] Raimondo J.V., Kay L., Ellender T.J., Akerman C.J. (2012). Optogenetic silencing strategies differ in their effects on inhibitory synaptic transmission. Nat. Neurosci..

[8] Mahn M., Prigge M., Ron S., Levy R., Yizhar O. (2016). Biophysical constraints of optogenetic inhibition at presynaptic terminals. Nat. Neurosci..

[9] Arenkiel B.R., Peca J., Davison I.G., Feliciano C., Deisseroth K., Augustine G.J., Ehlers M.D., Feng G. (2007). In Vivo Light-Induced Activation of Neural Circuitry in Transgenic Mice Expressing Channelrhodopsin-2. Neuron.

[10] Schjetnan A.G.P., Luczak A. (2011). Recording large-scale neuronal ensembles with silicon probes in the anesthetized rat. J. Vis. Exp..

[11] Vandecasteele M., M. S., Royer S., Belluscio M., Berényi A., Diba K., Fujisawa S., Grosmark A., Mao D., Mizuseki K. (2012). Large-scale Recording of Neurons by Movable Silicon Probes in Behaving Rodents. J. Vis. Exp..

[12] Vöröslakos M., Kim K., Slager N., Ko E., Oh S., Parizi S.S., Hendrix B., Seymour J.P., Wise K.D., Buzsáki G. (2022). HectoSTAR μLED Optoelectrodes for Large-Scale, High-Precision In Vivo Opto-Electrophysiology. Adv. Sci..

[13] McKenzie S., Huszár R., English D.F., Kim K., Christensen F., Yoon E., Buzsáki G. (2021). Preexisting hippocampal network dynamics constrain optogenetically induced place fields. Neuron.

[14] English D.F., McKenzie S., Evans T., Kim K., Yoon E., Buzsáki G. (2017). Pyramidal Cell-Interneuron Circuit Architecture and Dynamics in Hippocampal Networks. Neuron.

[15] Wu F., Stark E., Ku P.C., Wise K.D., Buzsáki G., Yoon E. (2015). Monolithically Integrated m LEDs on Silicon Neural Probes for High-Resolution Optogenetic Studies in Behaving Animals. Neuron.

[16] Kim K., Vöröslakos M., Seymour J.P., Wise K.D., Buzsáki G., Yoon E. (2020). Artifact-free and high-temporal-resolution in vivo opto-electrophysiology with microLED optoelectrodes. Nat. Commun..

[17] Valero M., Zutshi I., Yoon E., Buzsáki G. (2022). Probing subthreshold dynamics of hippocampal neurons by pulsed optogenetics. Science.

[18] Stark E., Koos T., Buzsáki G. (2012). Diode probes for spatiotemporal optical control of multiple neurons in freely moving animals. J. Neurophysiol..

[19] Royer S., Zemelman B.V., Barbic M., Losonczy A., Buzsáki G., Magee J.C. (2010). Multi-array silicon probes with integrated optical fibers: Light-assisted perturbation and recording of local neural circuits in the behaving animal. Eur. J. Neurosci..

[20] Robinson N.T.M., Descamps L.A.L., Russell L.E., Buchholz M.O., Bicknell B.A., Antonov G.K., Lau J.Y.N., Nutbrown R., Schmidt-Hieber C., Häusser M. (2020). Targeted Activation of Hippocampal Place Cells Drives Memory-Guided Spatial Behavior. Cell.

